# The association between early-life gut microbiota and childhood respiratory diseases: a systematic review

**DOI:** 10.1016/S2666-5247(22)00184-7

**Published:** 2022-11

**Authors:** Cristina Garcia-Maurino Alcazar, Veena Mazarello Paes, Yan Shao, Clarissa Oesser, Ada Miltz, Trevor D Lawley, Peter Brocklehurst, Alison Rodger, Nigel Field

**Affiliations:** a Institute for Global Health, University College London, London, UK; b Institute for Child Health, University College London, London, UK; c John Radcliffe Hospital, University of Oxford, Oxford, UK; d Host-Microbiota Interactions Laboratory, Wellcome Sanger Institute, Hinxton, UK; e Royal Free Hospital, Royal Free London NHS Foundation Trust, London, UK; f Institute of Applied Health Research, University of Birmingham, Birmingham, UK

## Abstract

Data from animal models suggest a role of early-life gut microbiota in lung immune development, and in establishing susceptibility to respiratory infections and asthma in humans. This systematic review summarises the association between infant (ages 0–12 months) gut microbiota composition measured by genomic sequencing, and childhood (ages 0–18 years) respiratory diseases (ie, respiratory infections, wheezing, or asthma). Overall, there was evidence that low α-diversity and relative abundance of particular gut-commensal bacteria genera (*Bifidobacterium*, *Faecalibacterium*, *Ruminococcus*, and *Roseburia*) are associated with childhood respiratory diseases. However, results were inconsistent and studies had important limitations, including insufficient characterisation of bacterial taxa to species level, heterogeneous outcome definitions, residual confounding, and small sample sizes. Large longitudinal studies with stool sampling during the first month of life and shotgun metagenomic approaches to improve bacterial and fungal taxa resolution are needed. Standardising follow-up times and respiratory disease definitions and optimising causal statistical approaches might identify targets for primary prevention of childhood respiratory diseases.

## Introduction

Childhood respiratory diseases, including respiratory infection, recurrent wheezing, and asthma, are important causes of morbidity and mortality in children and at ages thereafter. Up to 30% of children worldwide will develop at least one viral lower respiratory tract infection (vLRTI) during their first 2 years of life,^1^ mainly due to respiratory syncytial virus, and a third of these children will have subsequent recurrent wheezing episodes.^2^ Although asthma-like symptoms might be present before age 2 years, there are no reliable diagnostic tools to ascertain a diagnosis of asthma in children younger than 5 years.^3^, ^4^ Asthma prevalence from age 5 years to 14 years is estimated to be around 10%,^5^ making it the most prevalent chronic disease in childhood globally.^6^ Despite the huge health burden of vLRTIs, there are currently no widespread licensed primary prevention strategies for them or for asthma in children.^3^, ^7^ This absence of strategies is partly due to an incomplete understanding of disease pathogenesis—although host immune response seems to play an important role in susceptibility to both vLRTI and asthma.^8^, ^9^ It is now known that the innate and adaptive immune systems of individuals are influenced by their gut microbiota composition during the first year of life,^10^, ^11^ which might be a determinant of childhood respiratory disease aetiology.

Animal models have provided evidence that early-life gut microbiota composition might influence respiratory immunity and susceptibility to both asthma and respiratory infections.^12^, ^13^ This organ-level interaction is referred to as the gut–lung axis. Mechanistically, bioactive bacterial ligands and metabolites derived from the gut might enter the circulation to affect immune cell migration in the lung.^14^ Summarising available human observational evidence exploring the association between early-life gut microbiota composition and childhood respiratory diseases might help inform future intervention studies.

The gut microbiota is the largest and most diverse microbiota in the body, harbouring billions of bacteria (the predominant organisms), archaea, eukaryotes, and viruses.^15^ Gut microbiota colonisation starts at birth and is highly dynamic during the first years of life, stabilising after 1–3 years.^16^ Clinical, maternal, feeding, and environmental factors shape the early-life gut microbiota composition.^17^, ^18^ For example, infants delivered by caesarean section have a higher abundance of opportunistic pathogens during the neonatal period than do infants delivered by a vaginal birth.^18^ To a lesser extent, the same is true for infants who are not breastfed.^18^ Gut microbiota is usually measured by stool sample collection and can broadly be described in terms of diversity and abundance. Diversity describes the number of different taxa within a community. α-diversity refers to the number of taxa detected per sample, whereas β-diversity indicates the difference in composition between samples.^19^ More nuanced comparisons identify specific relative abundance of bacteria or fungi at different taxonomic levels.

In the past decade, genomic sequencing technologies and bioinformatic analytical tools have advanced considerably. Available platforms now allow the simultaneous sequencing of most or all genetic material present in stool samples,^20^ enabling an untargeted and more in-depth exploration of the gut microbiota composition and functional community dynamics.^21^ A widely used genomic sequencing technique is the amplicon approach, which sequences the ribosomal 16S rRNA gene and is highly conserved across all bacterial species. This sequencing technique enables resolution to the bacterial genus level.^22^ Shotgun metagenomic sequencing refers to the sequencing of all DNA present in a sample without targeting.^22^ This approach can readily resolve bacteria, fungi, viruses, and other microorganisms to strain level and can be used to infer gene functionality.^20^ However, both 16S rRNA gene sequencing and shotgun metagenomic sequencing involve multiple steps and complex technical challenges that can introduce measurement bias, including sample storage, DNA extraction, sample quality control, and bioinformatic pipelines.^19^, ^20^, ^23^ Moreover, studies combining epidemiological and genomic sequencing data could be subject to a range of limitations that might threaten legitimate inference.^20^

We systematically reviewed the existing literature to examine the association between gut microbiota composition during infancy (measured by genomic sequencing) and the subsequent development of respiratory disease during childhood.

## Method

A systematic literature review was done in accordance with the Preferred Reporting Items for Systematic Reviews and Meta-Analyses guidelines.^24^ The protocol was prospectively registered in the International Prospective Register of Systematic Reviews (CRD42020184094).

### Search strategy and selection criteria

We comprehensively searched five electronic databases (MEDLINE [Ovid interface], Embase, Web of Science, Scopus, and Cochrane) for articles published with the full text in English between Jan 1, 2010, and April 27, 2021. A start year of 2010 was selected to ensure all studies using genomic sequencing for gut microbiota characterisation were captured.^25^ Four broad search terms were considered: “infancy”, “intestine”, “microbiota”, and “respiratory disease”. An information specialist was consulted and the search strategy was refined with an iterative process on the basis of inclusion of key studies to optimise the selection of MeSH terms and keywords. Additional studies were identified by searching the references of relevant systematic reviews and included studies (appendix pp 2–3).

### Screening and data extraction

The first reviewer (CG-MA) screened all titles, abstracts, and full texts of shortlisted articles. A second reviewer (VMP) screened 10% of studies at each stage of the screening process and results were compared between reviewers to check agreement. Any disagreement was resolved with a third reviewer (CO). Data were extracted by the first reviewer. The second reviewer independently extracted information from 80% of included studies to ensure accuracy in reporting and to minimise reviewer bias.

### Strategy for data synthesis

Differences in microbiota diversity, relative abundance of bacteria or fungi taxa (at species, genus, or family level), and any measure of association with 95% CIs relative to either diversity or abundance were extracted and reported as the main result. Other gut microbiota parameters analysed and findings related to gut microbiota composition and respiratory disease were also extracted (appendix pp 8–10).

### Assessment of methodological quality

Critical appraisal was performed independently by two reviewers (CG-MA and VMP) to assess the quality of included studies and provide context for the interpretation of the findings. Studies were assessed with the Strengthening the Reporting of Observational Studies in Epidemiology (STROBE) metagenomics framework for reporting of metagenomic studies.^20^ A checklist to evaluate reporting of the main sources of bias in metagenomic studies was generated from the framework (appendix pp 15–17). Studies were also assessed with the Newcastle-Ottawa Scale (NOS) quality assessment scale for evaluation of bias and study design limitations.^26^ By use of defined thresholds, the results from the NOS were translated to the Agency for Health Research and Quality standards and studies were rated as good, fair, or poor (table 1; appendix pp 11–12).^38^ When studies included multiple analyses aimed at answering several research questions within the same study, quality assessments were only applied to the analyses relevant to this systematic review.

**Table 1:** Study characteristic and main results

	**Study participants; age at stool sample collection**	Technique of microbiota determination*	**Respiratory outcome; age at outcome evaluation; follow-up n/N (%)**	**Method of evaluation; outcome definition**	**Participants in each outcome group**	**Children with respiratory disease versus no respiratory disease**		**Adjustments for diversity; adjustments for relative abundance**	**Study quality**
						Diversity and relative abundance of taxa	Validation		
Arrieta et al (2015);^27^ nested case-control study	319 healthy, more than 35 weeks' gestation, singleton newborn babies; 3 months and 1 year	16S rRNA V3, Ilumina HiSeq, and Greengenes database (2006); validation with qPCR and 16S rRNA V3 amplicon	Atopic wheeze (as a proxy for later asthma);† 1 year; NA (loss to follow-up does not apply in case-control study designs)	Parental questionnaires at 3 months, 6 months, and 1 year of age; study clinical assessment at age 1 year; four phenotypes: atopy (positive prick test at age 1 year), wheeze (≥1 wheezing episodes in first year of life), atopy and wheeze, and control individuals (no asthma or atopy at age 1 year)	87 (27%) in atopy group; 136 (43%) in wheeze group; 22 (7%) in atopy and wheeze group; 74 (23%) control individuals	No difference in α-diversity (Shannon index) between the four phenotypes at ages 3 months or 1 year; relative abundance of bacteria taxa (genera): atopy and wheeze (compared with control individuals) showed lower FLRV and *Peptostreptococcus* (p>0·05) at age 3 months, atopy and wheeze (compared with control individuals) showed lower *Oscillospira* at age 1 year (p>0·05), and no differences between participants with wheeze and participants without wheeze	Validation with qPCR (for FLRV): individuals with atopy and wheeze have lower relative abundance of FLRV genera at age 3 months than control individuals (p<0.0001)	Unadjusted; unadjusted	Poor
Laursen et al (2015);^28^ cohort study	114 healthy, singleton children; 9 months and 18 months	16S rRNA V3, Ion Torrent, OneTouch, and Ion Personal Genome Machine systems; CLC Genomic workbench, Ribosomal database project classifier	Asthmatic bronchitis (cumulative prevalence); 3 years; at 3 years: 104/114 (91·2%)	Parental interviews when child was age 9 months, 18 months, and 3 years; cumulative prevalence (age 0–3 years) of diagnosed asthmatic bronchitis defined as squeaky and wheezing breathing in connection with cold or other viral infections in the respiratory system	19/104 (18%) had asthmatic bronchitis	No differences in α-diversity (Shannon Index) at ages 9 months or 18 months; relative abundance of bacteria taxa (genera): no differences at ages 9 months or 18 months (p>0·05 after correction for multiple testing)	NA	Unadjusted; unadjusted	Poor
Fujimura et al (2016);^29^ cohort study	298 children (all births); 130 at 1 month (neonatal) or‡ 168 at 6 months (infants)	16S rRNA V4, Illumina MiSeq, Greengenes database (2013), and Ribosomal Database Project classifier; fungal internal transcribed spacer region 2, UNITE database V6	Asthma; 4 years; at 4 years: 111/130 (85%, neonatal samples), 168/168 (100%, infant samples)	Parental interviews at months 1, 6, 12, 24, and 48, and clinical study visit at age 2 years; asthma according to parental-reported doctor diagnosis of asthma	39/279 (14%) had asthma; 17/111 (15%) were neonatal, 22/168 (13%) were infants	α-diversity not reported relative to asthma risk; bacterial β-diversity (PERMANOVA; R2=0·09, p<0·001) and fungal β-diversity (Bray–Curtis; PERMANOVA, R2=0·037, p=0·068) differed between clusters; relative abundance of bacteria taxa (genera): no differences in infant group (age 6 months), newborn babies with high risk of asthma at age 4 years (OR 1·09–10·3; p<0·05) had lower *Bifidobacterium, Lactobacillus Faecalibacterium*, and *Akkermansia* compared with low-risk asthma participants (adjusting for false discovery rate: B-H q<0·05); relative abundance of fungal taxa: newborn babies with high risk of asthma had lower *Malassezia* and higher *Candida* and *Rhodotorula* compared with low-risk asthma participants (adjusting for false discovery rate: B-H q<0·20)	NA	Unadjusted; unadjusted (data reported and individually adjusted for current breastfeeding, detectable cat allergen, detectable dog allergen, ever breastfed, female, first-born, maternal age, maternal education, maternal history allergic disease, birth delivery method, pets, and race)	Poor
Stiemsma et al (2016);^30^ nested case-control study	76 healthy, term (>35 weeks), singleton newborn babies; 3 months and 1 year	16S rRNA V3, Hiseq Ilumina, and Greengenes database (2006); validation with qPCR and 16S rRNA V3 amplicon	Asthma; 4 years; NA	Multiple questionnaires and clinical assessments by study physicians at ages 1 year and 3 years; cases defined as physician diagnosis of asthma by age 4 years or prescribed inhaled medications from ages 3–4 years and controls defined as negative for asthma or inhaled medication, wheezing, and atopy (based on standardised allergen skin prick testing at ages 1 year and 3 years)	39 (51%) with asthma; 37 (49%) matched healthy controls	No differences in α-diversity (Shannon index) or β-diversity at ages 3 months or 1 year; relative abundance of bacteria taxa: individuals with asthma showed lower Clostridiales (class; log2FC 1–2; p=0·035) and *Lachnospira* (genera; log2FC 1–2; p=0·098), higher *Clostridium neonatale* (species; log2FC 1–2; p=0·076), Clostridiaceae (family; p=0·005), and Firmicutes (phylum; log2FC 1–3; p=0·035) than did control individuals at age 3 months, individuals with asthma showed higher Lachnospiraceae (family; p=0·032) and *Rothia* (genera; p=0·003) than did control individuals at age 1 year	Validation with qPCR: individuals with asthma showed lower relative abundance of *Lachnospira* at age 3 months (p=0·008) and lower *Clostridium neonatale* at age 1 year (p=0·02)	Matched on gender, birth delivery method, feeding practices, antibiotic exposure; matched on gender, birth delivery method, feeding practices, antibiotic exposure	Good
Arrieta et al (2018);^31^ nested case-control study	97 healthy newborn babies; 3 months	16S rRNA V4, Miseq Ilumina, Greengenes database (2006), 18S rRNA V4 (fungi), SILVA database (2013); validation with qPCR	Atopic wheeze; 5 years; NA	Parental interviews; cases defined as maternally reported wheeze in the previous 12 months at age 5 years and positive skin prick test response and controls defined as random sample of children with no previous history of wheeze and no evidence of atopy at age 5 years	27 (28%) with atopic wheeze, 70 (72%) healthy controls	No differences in α-diversity (Chao1 index) or β-diversity, bacterial or fungal; relative abundance of bacteria taxa (genera): children with atopic wheeze had low *Bifidobacterium* (log2FC>4; p<0·001) and high *Streptoccocus* (log2FC 2–4; p=0·044) and *Veillonella* (log2FC 2–4; p=0·031), adjusted for false discovery rate at age 3 months; relative abundance of fungal taxa (genera): children with atopic wheeze had higher *Pichia Kudriavzevii* at age 3 months (log2FC 2–6; p<0·01)	Validated increase in *Pichia kudriavzevii* species using qPCR at 3 months in children with atopic wheeze compared with healthy controls	Unadjusted; adjusted for antibiotic use during pregnancy or during first year of life, antibiotic duration, birth delivery method, household potable water, number of respiratory tract infections during first year of life, eosinophilia at age 7 months, number of diarrhoeal episodes during first year of life	Fair
Stockholm et al (2018);^32^ cohort study	690 newborn babies (all births); 1 week, 1 month, and 1 year	16S rRNA V4, Illumina Miseq, and Greengenes database (2013)	Asthma; 5 years; at 5 years: 648/690 (94%)	Clinical visits at age 1 week, ages 1, 3, 6, 12, 18, 24, 30, and 36 months, and yearly thereafter (including during acute respiratory episodes); asthma diagnosis if all of 5 episodes of lung symptoms within 6 months (at least for 3 consecutive days), exercise-induced symptoms, prolonged nocturnal cough, or persistent cough outside of common colds, need for intermittent rescue use β2-agonist, and response to 3 months of inhaled steroids and relapse after stopping	60/648 (9%) had asthma	No differences in α-diversity (Shannon and Chao1 indices) at any timepoint; no differences in β-diversity at ages 1 week and 1 month; β-diversity at 1 year in individuals with asthma *vs* individuals without asthma (PERMANOVA; *F*=3·4, R2=0·6%, p=0·003); relative abundance of bacteria taxa (genera): no differences at ages 1 week or 1 month; for the 20 most abundant bacterial genera, individuals with asthma had lower *Roseburia* (median relative abundance 0·27% *vs* 0·66%; p=0·042), *Alistipes* (median relative abundance 0·04% *vs* 0·35%; p=0·002), and *Flavonifractor* (0·05% *vs* 0·07%; p=0·002) and higher *Veillonella* (0·94% *vs* 0·29%; p=0·035) at age 1 year than had controls; diversity and relative abundance differences were influenced by children born to mothers with asthma (effect modifier; p=0·011)	NA	Adjusted for birth delivery method, duration of exclusive breastfeeding, older sibling hospitalisation after birth, and antibiotic use; unadjusted	Good
Reyman et al (2019);^33^ cohort study	120 healthy, term (>37 weeks) newborn babies; 1 week	16S rRNA V4, llumina MiSeq, Naive Bayesian Ribosomal Database Project classifier (version 2.2), and SILVA database (2012); validation with qPCR	Respiratory infection (cumulative incidence); 1 year; at 1 year: 118/120 (98%)	Structured interview and questionnaire; respiratory infection defined as fever (>38·0°C) and any of cough, wheezing, dyspnoea, earache, or malaise (events in the first year of life were summed up to a cumulative number and categorised into 0–2 *vs* 3–7)	77 (65%) 3–7 respiratory infections; 41 (35%) with 0–2 respiratory infections (considered healthy)	Diversity not reported relative to respiratory infection; relative abundance of bacterial taxa (genera): children with more respiratory infections (those with 3–7 compared with those with 0–2) had lower *Bifidobacterium* (log2FC 2·1; p=0·049) and highe*r Klebsiella* (log2FC 3·2; p=0·007) and *Enterococcus* (log2FC 2·8; p=0·009) at age 1 week	Validation with qPCR: children with 3–7 respiratory infections had higher relative abundance of *Enterococcus* spp (p=0·015) than did children with 0–2 respiratory infections	NA; adjusted for birth delivery method	Good
Galazzo et al (2020);^34^ cohort study	440 healthy, term newborn babies and parents with atopic disease; 5 weeks, 3·3 months, 5·3 months, and 7·8 months	16S rRNA V3, Illumina MiSeq, and Greengenes database (2011)	Asthma; 6–11 years; at 6–11 years: 292/440 (66%)	Clinical visit; parent-reported doctor diagnosis of asthma in combination with any indicative symptoms in the past 12 months (wheezing, shortness of breath, nocturnal awakening due to symptoms; included lung function testing)	Not reported	No differences in α-diversity (Shannon index); relative abundance of bacteria taxa (genera): children with asthma showed, at all ages, lower *Lachnobacterium*, *Lachnospira*, and *Dialister* (p<0·001) than children without asthma	NA	Adjusted for breastfeeding, age at introduction to solid food, birth delivery method, treatment (placebo *vs* probiotic), birthweight, sex, mother or father with atopic dermatitis, >2 older siblings, and pets; adjusted for breastfeeding, age at introduction to solid food, birth delivery method, treatment (placebo *vs* probiotic), birthweight, sex, mother or father with atopic dermatitis, >2 older siblings, and pets	Good
Boutin et al (2020);^35^ cohort study	837 healthy, term (>35 weeks), singleton newborn babies; 3 months	16S rRNA V4, HiSeq Ilumina, and Greengenes database (2013)	Recurrent wheeze and atopic wheeze; 1 year; 659/837 (79%) after 1 year	Questionnaires and clinical assessments by study physicians at ages 1, 3, and 5 years; recurrent wheezing defined as ≥2 episodes of wheezing in the first year of life, healthy control individuals did not have asthma, recurrent wheeze, atopic dermatitis, atopy, or allergic sensitisation at ages 1, 3, and 5 years	142/659 (21%) had recurrent wheeze; 45/659 (6%) had atopic wheeze; 16/659 (2%) had both (included in recurrent wheeze and atopic wheeze groups)	Children with recurrent wheeze and atopic wheeze had lower α-diversity at age 3 months than individuals without; increased α-diversity at age 3 months was protective of recurrent wheeze (OR 0·75 [0·6–0·95]; p=0·007) and atopic wheeze (OR 0·55 [0·1–0·90]; p=0·016); relative abundance of bacterial taxa (analyses aimed at identifying only decreased bacteria genus): children with recurrent wheeze had lower *Faecalibacterium*, *Lachnospira*, *Coprococcus*, and *Oscillospira* at age 3 months than did healthy children and children with atopic wheeze showed lower *Faecalibacterium*, *Lachnospira*, *Coprococcus*, *Roseburia*, *Blautia*, *Parabacteroides*, and *Ruminococcus* at age 3 months than did healthy children	NA	Unadjusted; unadjusted (machine learning predictive methods)	Poor
Patrick et al (2020);^36^ cohort study	917 healthy, term (>35 weeks), singleton newborn babies; 3 months and 1 year	16S rRNA V4, Hiseq Ilumina, and Greengenes database (2013)	Asthma; 5 years; outcome evaluated in those with stool sample processed at 1 year: 570/917 (62%)	Multiple questionnaires and clinical assessments by study physicians at ages 1, 3, and 5 years; diagnosis of asthma based on questionnaire data, clinical history, and medical examination	63/570 (11%) had asthma	At age 1 year, children with asthma showed decreased α-diversity (Chao1 index) compared with children without asthma; having increased α-diversity at age 1 year protected from asthma (OR 0·68 [0·46–0·99]; p=0·046); relative abundance of bacterial taxa (only summarising those with at least 1·5 log2FC): at age 1 year, children with asthma had lower *Faecalibacterium prausnitzii* (log2FC −1·57 to 1·77*)*, *Ruminococcus bromii* (log2FC −2·07*)*, and Rikenellaceae (family) (log2FC −2·59) and higher *Dialister* (genus) (log2FC 2·04; false discovery rate p<0·05) than did children without asthma§	NA	Adjusted for antibiotic use at age 1 year, race, birth delivery method, older siblings, sex, birthweight, prenatal atopy, breastfeeding at age 6 months, tobacco smoke exposure in first year of life, NO_2_ exposure during first year of life, season of birth, living area (urban or rural); adjusted for antibiotic use at age 1 year, race, birth delivery method, older siblings, sex, birthweight, prenatal atopy, breastfeeding at age 6 months, tobacco smoke exposure in first year of life, NO_2_ exposure during first year of life, season of birth, living area (urban or rural), sequencing batch, and study centre	Good
Depner et al (2020);^37^ cohort study	720 newborn babies; 2 months and 1 year	16S rRNA V4, Illumina MiSeq, and Greengenes database (2013); fungal internal transcribed spacer region 1, UNITE dynamic database (2010)	Asthma; 6 years; at 6 years: 626/720 (87%)	Parent-reported doctor diagnosis of asthma at least once or recurrent diagnoses of obstructive bronchitis or asthmatic bronchitis; atopic and non-atopic asthma defined as the presence or absence of concomitant sensitisation to inhalant allergens (seasonal or perennial) with specific IgE concentrations higher than 0·7 IU/mL–1 at age 6 years (lung function measured by spirometry at age 6 years)	53/626 (9%) had asthma	Reported EMA¶ as a proxy for α-diversity (positive correlation [*r*=0·70] for number of different bacteria genera); children with asthma had a difference in EMA at age 2 months and lower EMA at age 1 year than did children without asthma; relative abundance of bacteria taxa (genera): children with asthma had, at age 2 months, lower *Bacteroides* and *Parabacteroides* and higher *Enterococcus* than did children without asthma; having a high relative abundance of *Bacteroides* and *Parabacteroides* and low relative abundance of *Enterococcus* at age 2 months protected from both atopic and non-atopic asthma (OR 0·68 [0·49–0·95]; p=0·024); children with asthma showed, at age 1 year, lower *Roseburia, Ruminococcus*, and *Faecalibacterium* than did children without asthma; having higher relative abundance of *Roseburia, Ruminococcus*, and *Faecalibacterium* at age 1 year protected from non-atopic asthma (OR 0·62 [0·39–1·00]; p=0·048; association fully explained by EMA)	NA	Unclear if adjusted (collected clinical variables including birth delivery method, breastfeeding, antibiotic use, gestation age, birthweight, Apgar score, parental history of atopy, farm exposure, pets, smoke exposure, number of siblings, and parental education)	Good

API= Asthma Predictive Index. B-H=Benjamini-Hochberg. EMA=estimated microbiome age. FLRV=*Faecalibacterium, Lachnospira, Rothia, Veillonella*. Log2FC=log2 fold change. NA=not available. OR=odds ratio. qPCR=quantitative PCR.

* Target, sequencing platform, pipeline, and database.

† Children were followed up for 3 years. At age 3 years children had a clinical visit to predict asthma development between ages 6 years and 11 years using the API. The atopy and wheezing group at age 1 year were 5·4 times more likely to have a positive API score at age 3 years compared with the wheeze only group at 1 year.

‡ All other studies used and instead of or (participants were followed up and samples were collected at different timepoints).

§ Findings extracted from supplementary materials. Findings reported in main paper were that patients with asthma and antibiotic use at age 1 year had relative decreased abundance of *Faecalibacterium prausnitzii*, *Roseburia* (genera), and *Ruminococcus bromii* and increased abundance of *Clostridium perfringens* (false discovery rate p<0·05).

¶ Random forest analysis (machine learning) was used to estimate the healthy age of gut microbiota sampled at ages 2 months and 1 year in 133 healthy individuals (no diarrhoea, wheezing, or asthma in the first year of life).

## Results

4759 titles and abstracts were screened and 4648 of these studies were excluded. Reasons for exclusion included non-primary research, animal studies, in-vitro studies, or studies in which exposure or outcome were not relevant to the research question. After a full-text review of the remaining 111 studies, 11 were included (figure). Agreement between the first and second reviewer was high (κ=0·98).

**Figure:**
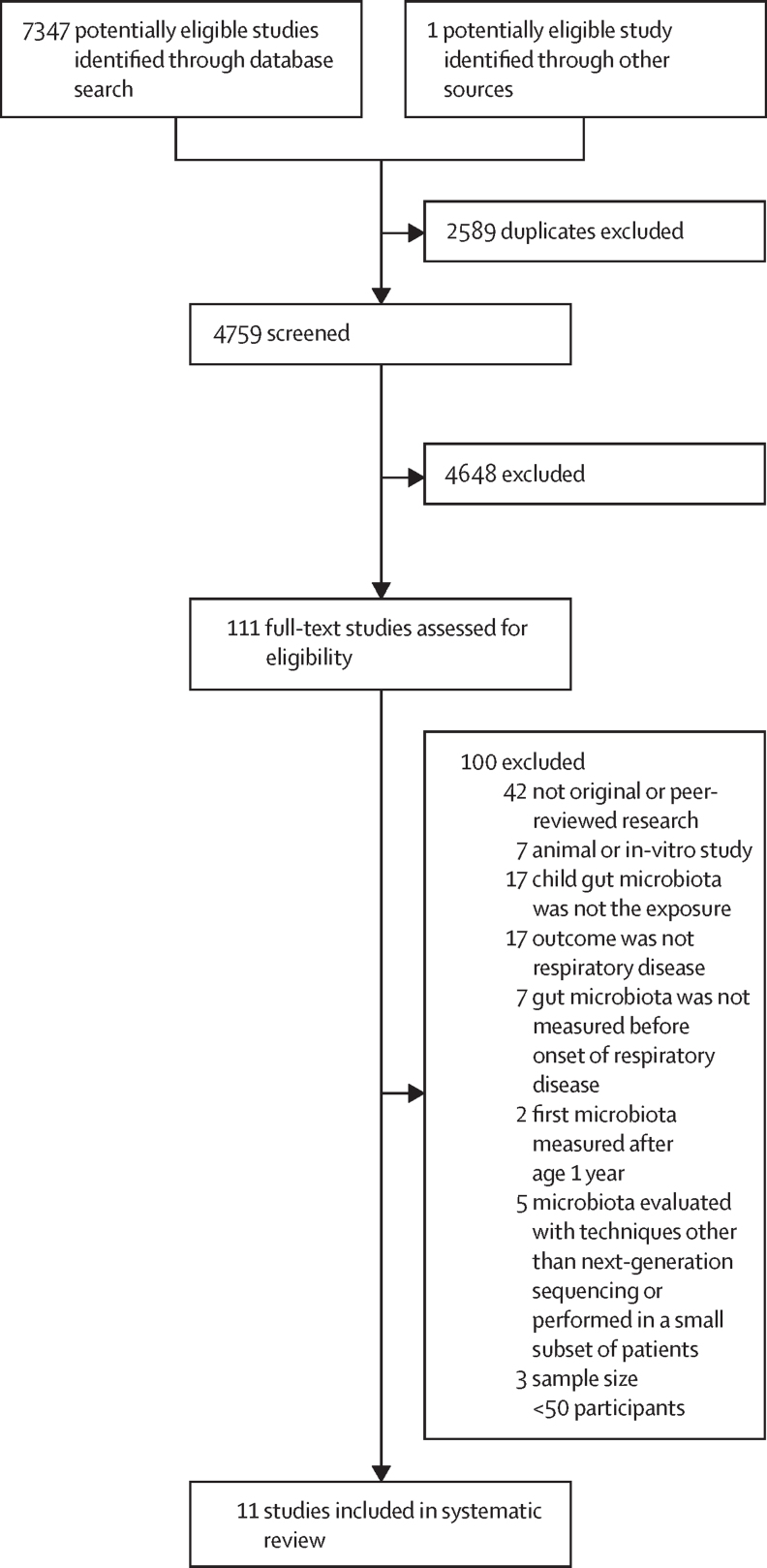
Study selection

Of the 11 included studies, eight were cohort studies^28^, ^29^, ^32^, ^33^, ^34^, ^35^, ^36^, ^37^ and three were nested case-control studies (table 1).^27^, ^30^, ^31^ Nine studies were done in urban areas in Europe or the USA and two studies were done in rural areas of Europe and Ecuador.^31^, ^37^ Four studies were from the same research group and based on the same birth cohort, with potential overlap of participants.^27^, ^30^, ^35^, ^36^ Sample size ranged from 76 participants to 917 participants, with a median of 319. Eight studies^27^, ^28^, ^29^, ^30^, ^34^, ^36^, ^37^ characterised the gut microbiota composition at more than one timepoint, but only three studies collected stool samples in the neonatal period (first month of life).^29^, ^32^, ^33^ All studies used amplicon sequencing targeting the 16S rRNA (V3 or V4 regions) gene for gut microbiome determination. Three studies included evaluation of the presence of fungi.^29^, ^31^, ^37^ Respiratory disease status was ascertained on the basis of parental reported symptoms or reported doctor diagnosis of respiratory disease in all but one study, in which the outcome was ascertained with clinical visits during acute respiratory episodes.^32^ Two studies mentioned that lung function tests were done,^32^, ^34^ but it was unclear how these tests were integrated into respiratory disease definitions.

Four of the 11 included studies were classified as poor quality,^27^, ^28^, ^29^, ^35^ one was classified as fair quality,^31^ and six were classified as good quality (table 1, appendix pp 11–13).^30^, ^32^, ^33^, ^34^, ^36^, ^37^ Study design limitations were mainly due to potential selection bias or unadjusted confounders. Most studies selected participants on the basis of completed follow-up or availability of stool samples, whereas only three studies^31^, ^32^, ^36^ compared participants with and without stool availability or complete follow-up, showing low probability of selection bias. No studies presented hypothesised a-priori causal pathways regarding variables associated with exposures or outcomes (potential confounders),^39^ and three studies did not mention adjusting for confounding factors.^27^, ^28^, ^35^ The other eight studies adjusted for some potential confounders, such as breastfeeding or delivery method. However, other important potential confounders, such as socioeconomic status or smoking exposure,^40^ were not accounted for in most analyses of these eight studies.

Regarding the STROBE metagenomics checklist (appendix pp 14–17), all included studies provided at least partial methods for specimen collection, storage, and DNA extraction, although six studies^27^, ^30^, ^31^, ^34^, ^35^, ^36^ did not clearly report time between sample collection and freezing. Six studies^27^, ^29^, ^30^, ^31^, ^32^, ^33^ explicitly reported use of negative control samples to exclude contamination. Sequencing methods and bioinformatic pipelines were at least partly reported by all included studies. Seven studies amplified the V4 hypervariable region of the 16S rRNA gene, and four studies amplified the V3 region of the 16S rRNA gene.^27^, ^28^, ^30^, ^34^ All studies except one^28^ used Illumina HiSeq or MiSeq sequencing platforms. Bioinformatic pipelines for data cleaning varied between studies. Four used Mothur^27^, ^30^, ^31^, ^32^ and six used QIIME or QIIME2.^29^, ^31^, ^33^, ^35^, ^36^, ^37^ All but one study^33^ used a version of the Greengenes database (2006 or 2013) as a reference for defining bacterial operational taxonomic units.

The statistical analyses used to establish the association between diversity and relative abundance of taxa and respiratory disease included univariate statistical tests,^27^ correlation matrices adjusted for false discovery rates,^28^ DESeq2,^30^, ^31^, ^36^ multivariate regression models adapted to microbiome data (eg, MaAsLin),^41^ and predictive machine learning approaches.^33^, ^35^ Most included studies compared microbiota diversity or raw relative abundance of taxa between participants with and without respiratory disease, whereas others used clustering approaches based on microbiota composition.^29^, ^32^, ^34^, ^37^ Four studies used quantitative PCR as a confirmatory assay^27^, ^30^, ^31^, ^33^ and one performed next-generation sequencing in a subset of 20 samples.^33^ Two studies used experimental models (animal or in-vitro experiments) to validate observational findings.^27^, ^29^ Four studies^27^, ^29^, ^31^, ^37^ evaluated gut microbiota functionality by measuring concentrations of short chain fatty acids and bacterial metabolites^42^ in stool samples (appendix pp 8–10). Only two studies considered a-priori power calculations; however, no specific estimations of power were reported.^30^, ^33^ Although cross-sectional studies were excluded, the potential for reverse causality was identified in two of the included studies, in which respiratory disease was assessed before, at the same time as, or after one of the timepoints for stool collection.^27^, ^28^

Study results have been summarised and stratified by type of respiratory disease and age at stool sample collection^17^ (Table 1, Table 2, Table 3). Two groups of studies were identified on the basis of respiratory outcome definition, including whether atopy was considered and age at outcome determination.^3^ The first group included nine studies that explored atopic wheeze or asthma^27^, ^29^, ^30^, ^31^, ^32^, ^34^, ^35^, ^36^, ^37^ (table 2), of which four studies followed up participants to age 5 years,^31^, ^32^, ^35^, ^36^ one study followed up participants to age 6 years,^37^ and one study followed up participants between age 6 years and age 11 years.^34^ The second group included studies exploring respiratory infections or studies exploring wheezing in the context of respiratory infections (first year of life), referred to as “respiratory infection or wheezing” (table 3).^27^, ^28^, ^33^, ^35^ Two studies explored wheeze both with and without atopy (defined as positive prick test) at age 1 year and were included in both groups.^27^, ^35^

**Table 2:** Studies exploring asthma or atopic wheeze

	**Age at gut microbiota determination**	**α-diversity (respiratory disease *vs* no respiratory disease)**	**Relative abundance of bacteria taxa (or fungal taxa) in respiratory disease versus no respiratory disease**	**Age of participants at respiratory disease determination (outcome)**	**AHRQ rating**
Fujimura et al (2016)^29^	≤1 month	Not reported	Lower *Bifidobacterium*, *Lactobacillus*, *Faecalibacterium*, and *Akkermansia*; lower Malassezia; higher *Candida* and *Rhodotorula*	4 years (high risk of asthma)	Poor
Stockholm et al (2018)^32^	≤1 month	No difference	No difference	5 years (asthma)	Good
Arrieta et al (2015)^27^*	3 months	No difference	Lower *Faecalibacterium*, *Lachnospira*, *Rothia*, *Veillonella*, and *Peptostreptococcus*	1 year (atopic wheeze)	Poor
Boutin et al (2020)^35^*	3 months	α-diversity decreased	Lower *Faecalibacterium*, *Lachnospira*, *Coprococcus*, *Roseburia*, *Blautia*, *Parabacteroides*, and *Ruminococcus*	1 year (atopic wheeze)	Poor
Stiemmsa et al (2016)^30^*	3 months	No difference	Lower Clostridiales and *Lachnospira*; higher *Clostridium neonatale* (species), Clostridiaceae (family), and Firmicutes (phylum)	4 years (asthma)	Good
Arrieta et al (2018)^31^	3 months	No difference	Lower *Bifidobacterium*; higher *Streptococcus*, *Veillonella*, and *Pichia kudriavzevii*	5 years (atopic wheeze)	Fair
Arrieta et al (2015)^27^*	1 year	No difference	Lower *Oscillospira*	1 year (atopic wheeze)	Poor
Stiemmsa et al (2016)^30^*	1 year	No difference	Lower *Clostridium neonatale*; higher Lachnospiraceae and *Rothia*	4 years (asthma)	Good
Stockholm et al (2018)^32^	1 year	No difference	Lower *Roseburia*, *Alistipes*, and *Flavonifractor*; higher *Veillonella*	5 years (asthma)	Good
Patrick et al (2020)^36^*	1 year	α-diversity decreased	Lower *Faecalibacterium prausnitzii*, *Ruminococcus bromii*, and Rikenellaceae (family); higher *Dialister*	5 years (asthma)	Good
Depner et al (2020)^37^	1 year	α-diversity decreased	Lower *Faecalibacterium*, *Roseburia*, and *Ruminococcus*	6 years (non-atopic asthma)	Good

* Studies from the same cohort. AHRQ=Newcastle-Ottawa Quality assessment for cohort and case-control studies converted to the Agency for Healthcare Research and Quality scale. One paper^29^ did not report results independently by time of stool sample collection, but the authors reported consistent decreases in relative abundance of certain bacteria genera in gut microbiota (*Lachnobacterium, Lachnospira,* and *Dialister*) at all timepoints examined (5 weeks, 3·3 months, 5·3 months, and 7·8 months) in children who developed asthma (parent-reported doctor diagnosis of asthma at age 6–11 years) compared with children who did not develop asthma.

**Table 3:** Studies exploring respiratory infections or studies exploring wheezing in the context of respiratory infections

	**Age at gut microbiota determination**	**Diversity (respiratory disease vs non-respiratory disease)**	**Relative abundance of bacteria taxa (fungal taxa) in respiratory disease versus non-respiratory disease**	**Age of participants at respiratory disease determination (outcome)**	**AHRQ rating**
Reyman et al (2019)^33^	≤1 month	Not reported	Lower *Bifidobacterium*; higher *Klebsiella* and *Enterococcus*	1 year (cumulative incidence of respiratory infections)	Good
Arrieta et al (2015)^27^	3 months	No difference	No difference	1 year (wheezing)	Poor
Boutin et al (2020)^35^	3 months	α-diversity decreased	Lower *Faecalibacterium, Lachnospira, Coprococcus,* and *Oscillospira*	1 year (recurrent wheezing)	Poor
Laursen et al (2015)^28^	9 months	No difference	No difference	3 years (wheezing in respiratory infection context)	Poor
Arrieta et al (2015)^27^	1 year	No difference	No difference	1 year (wheezing)	Poor

AHRQ=Newcastle-Ottawa Quality assessment for cohort and case-control studies converted to the Agency for Healthcare Research and Quality scale.

### Asthma and atopic wheezing

Of the seven studies^27^, ^30^, ^31^, ^32^, ^35^, ^36^, ^37^ that explored the direct association between α-diversity and asthma or atopic wheeze, two reported that higher gut microbiota α-diversity in the first year of life, compared with lower α-diversity, was significantly associated with not having atopic wheeze at age 1 year, and not having asthma at ages 5 and 6 years.^35^, ^36^, ^37^ The other five studies observed no association. Three studies used an alternative measure for describing overall gut microbiota composition. These studies explored gut microbial maturity on the basis of bacterial taxa compositional changes over time in a subset of healthy participants and compared this microbiota maturity with that of participants who developed childhood respiratory disease (appendix pp 7–10). One study used this method as a proxy for diversity, as they were positively correlated.^37^ One study reported that increased gut microbiota maturity at age 5 weeks, compared with decreased gut microbiota maturity, was associated with high risk of asthma in participants aged 6–11 years,^34^ and two studies reported an association between an immature gut microbiota at age 12 months and increased risk of asthma at ages 5 years and 6 years.^32^, ^37^

Although results varied between studies, overall there was evidence of a low relative abundance of the genera *Bifidobacterium*^29^, ^31^ in stools collected at ages 1 month and 3 months and low abundance of the genera *Faecalibacterium*,^27^, ^35^, ^36^, ^37^ *Roseburia*,^32^, ^35^, ^37^ and *Ruminococcus*^35^, ^36^, ^37^ in stools collected at ages 3 months and 1 year being associated with asthma and atopic wheeze at ages 1–6 years. Low relative abundance of *Lachnospira*^27^, ^30^, ^35^ at age 3 months but increased abundance of *Lachnospira* at age 1 year^30^, ^32^ was also associated with asthma and atopic wheeze at ages 1–6 years. One study showed low relative abundance of *Veillonella*^27^ at age 3 months was associated with atopic wheeze at age 1 year, whereas two studies reported that high relative abundance of *Veillonella* at ages 3 months and 1 year was associated with asthma and atopic wheeze at age 5 years.

Three studies^29^, ^31^, ^37^ explored the association between fungi and asthma. One study sequenced the conserved fungal marker genes, including the *18S rRNA* gene,^31^ and two studies sequenced the nuclear ribosomal Internal Transcribed Spacer (*ITS1* and *ITS2*).^29^, ^37^ High relative abundance of *Candida* and *Rhodotorula* and low abundance of *Malassezia* taxa measured at age 1 month in one study,^29^ and an increase of *Pichia kudriavzevii* at age 3 months measured in another,^34^ were associated with asthma and atopic wheeze at ages 4–5 years. The third study found no associations between fungal maturity and asthma at age 6 years.^37^

### Respiratory infections and wheezing

Four studies were included in the respiratory infections and wheezing group, in which respiratory disease definitions were highly heterogeneous. One study evaluated respiratory infections,^33^ another study evaluated wheezing in the context of respiratory infection,^28^ and two studies evaluated non-atopic wheeze^27^, ^35^ at age 1 year. Three studies^27^, ^28^, ^35^ explored the association between α-diversity and respiratory infections or wheezing and one study reported that high α-diversity in the first year of life was associated with reduced recurrent wheezing at age 1 year.^35^ The other two studies did not find an association between α-diversity and respiratory infections or wheezing. Two studies showed no association between relative abundance of species measured at ages 3, 9, and 12 months and wheezing at ages 1–3 years.^28^, ^31^ One study showed a lower relative abundance of *Bifidobacterium* and higher abundance of *Klebsiella* and *Enterococcus* at age 1 week in children with higher cumulative incidence of respiratory infections at age 1 year than for children with lower cumulative incidence of respiratory infections.^33^ No study specifically explored vLRTI as an outcome.

## Discussion

To our knowledge, this systematic review is the first to consider the association between early-life gut microbiota and childhood respiratory diseases, including respiratory infections, with a focus on genomic sequencing to measure the gut microbiota. Large studies (>700 participants) reported high α-diversity as being protective of asthma and wheezing.^35^, ^36^, ^37^ Overall, there was evidence of low relative abundance of *Bifidobacterium*^29^, ^31^, ^33^ in stools collected before age 3 months being associated with respiratory infections at age 1 year and asthma at ages 4–5 years. Generally, low abundance of the genera *Faecalibacterium*,^28^, ^35^, ^36^, ^37^ *Roseburia*,^32^, ^35^, ^37^ and *Ruminococcus*,^35^, ^36^, ^37^ in stool samples collected at ages 3–12 months were associated with asthma and atopic wheeze at ages 1–6 years. However, there were important study limitations, including heterogeneous outcome definitions and follow-up times, residual confounding, small sample sizes, and heterogeneous bioinformatic and statistical approaches, with most studies not reporting effect estimates.

A previous systematic review by Zimmerman and colleagues^25^ in 2019 assessed the association between gut microbiota and atopy, including asthma. Study inclusion was not restricted by method of microbiota determination, and 11 studies that independently reported wheezing or asthma as their outcome were included. Four of those studies were also included in this systematic review.^27^, ^29^, ^30^, ^31^ The other seven studies were excluded, as gut microbiota determination was not done with genomic sequencing. Instead, these seven studies used culture,^43^, ^44^ PCR testing targeting five bacteria,^45^, ^46^ or denaturing gradient gel electrophoresis.^47^, ^48^, ^49^ Zimmerman and colleagues summarised study results at a bacterial family taxa resolution. They concluded that high relative abundance of Bacteroidaceae, Clostridiaceae, and Enterobacteriaceae and low relative abundance of Bifidobacteriaceae and Lactobacillaceae were associated with the development of allergic sensitisation, eczema, or asthma.

All studies included in this systematic review used 16S rRNA gene amplicon sequencing, allowing a relatively untargeted starting point for exploring the gut microbiome and permitting us to summarise and compare study results at a bacterial genera level. Some studies reported that high relative abundance of non-commensal gut bacteria such as *Klebsiella* and *Enterococcus* at age 1 week^33^ was associated with respiratory infections at age 1 year, that high relative abundance of *Streptococcus* at age 3 months was associated with atopic wheeze at age 5 years, and that high relative abundance of *Rothia*^30^ or *Dialister*^36^ at age 1 year was associated with asthma at ages 4–5 years. However, study results seem to show more consistency with regard to low relative abundance of particular gut-commensal bacteria genera, such as *Bifidobacterium*,^29^, ^31^ *Faecalibacterium*,^27^, ^29^, ^35^, ^36^ *Ruminococcus*,^35^, ^36^, ^37^ or *Roseburia*,^35^, ^37^ in the first 1–12 months of life being associated with respiratory disease.

The genus *Bifidobacterium* constitutes one of the most abundant bacteria in the gut of children during the first 4 months of life^50^, ^51^ and has been shown to modulate the systemic immune response of individuals through surface-associated molecules and microbiota-derived metabolites both in vitro and in vivo.^52^ Specific *Bifidobacterium* spp have been shown to affect respiratory disease susceptibility in mouse models of asthma and respiratory infection.^12^, ^13^, ^53^ A 2020 study showed that gut colonisation with *Bifidobacterium infantis* regulates the equilibrium between Th1 and Th2 responses, reducing symptoms of atopic asthma in an induced mouse model.^12^ Another study reported that, when challenged with influenza virus, mice with higher gut abundance of *Bifidobacterium* and *Bacteroides* showed increased influenza survival through an enhanced CD8 T-cell and well regulated macrophage response than mice with lower gut abundance, preventing excessive airway neutrophil influx.^13^

The bacteria genera *Faecalibacterium*, *Ruminococcus*, *Lachnospira*, *Roseburia*, and *Veillonella* correspond to the clostridia class, which has been described in the guts of children from around 4–6 months of age, coinciding with weaning off breastmilk.^16^ *Ruminococcus* (specifically *Ruminococcus gnavus*) and *Roseburia* (specifically *Roseburia inulinivorans*) have been described as signature taxa in infants aged 12 months.^16^ Potential immune-modulation mechanisms have been described for *Roseburia* and *Faecalibacterium,* which produce butyrate, a bacterial metabolite with anti-inflammatory properties in animal and in-vitro models.^42^ Although one study included in this systematic review showed that inoculating germ-free mice with *Lachnospira*, *Veillonella*, *Faecalibacterium*, *and Rothia* improved airway inflammation in the adult progeny of these mice,^27^ little is known about the mechanistic role of these bacteria in respiratory disease.

Comparison of studies exploring respiratory infections and wheezing episodes in children younger than 5 years is challenging because of the inconsistent definitions for upper and lower viral respiratory infections and recurrent wheezing in the literature and clinical guidelines, and the difficulty in diagnosing asthma at ages 0–6 years.^54^ Correctly classifying wheezing phenotypes could be crucial as they seem to have divergent underlying pathophysiology, shown by the variability in response to different treatments (eg, steroids or β2 agonists).^55^ In turn, these phenotypes might be linked to different early gut microbiota compositions. We stratified results by respiratory disease type but still found that outcome definitions were inconsistent within groups, were mostly ascertained by parental interviews (with potential recall bias), and follow-up of children only went beyond age 5 years in two asthma studies. Wheezing in children younger than 5 years was used in most studies as a proxy for asthma, with some studies considering atopy and other studies not considering atopy. However, wheezing is a symptom and not a disease,^55^ so the results of these studies should be compared with caution and the measurements of outcomes are at risk of misclassification bias.

Only two studies included in this systematic review contained respiratory infections per se in their outcome definition.^28^, ^33^ The largest and most robust study showed that low relative abundance of *Bifidobacterium* and high relative abundance of *Klebsiella* and *Enterococcus* in stool samples collected at age 1 week were associated with a higher number of respiratory infections evaluated at age 1 year compared with those with low numbers of respiratory infections.^33^ As such, a considerable knowledge gap exists with respect to respiratory infections.

Neonatal gut microbiota composition is known to influence subsequent colonisation patterns, which might also affect subsequent microbiota and immune crosstalk and development in the long term.^13^ However, only three studies included in this systematic review collected stool samples in the neonatal period (first month of life),^28^, ^35^, ^37^ even though this age has been highlighted as an important time period for potential microbiota-altering interventions.^56^, ^57^ Although Zimmerman and colleagues^25^ reported consistency regarding study results in stool samples collected in the neonatal period in six wheezing or asthma studies, as our review only included three studies with neonatal stool samples, we found more consistency in results from studies with stool samples collected at ages 3–12 months. Gut microbiota composition beyond the first year of life might also be important, although careful assessment of timing of respiratory disease ascertainment will be needed to avoid reverse causality.

Despite relatively good reporting of laboratory methods and bioinformatic pipelines, these methods and pipelines were heterogeneous, important information was sometimes missing, and potential procedure-specific bias was barely discussed. For example, six studies did not clearly report time between sample collection and freezing and no studies mentioned freeze –thaw cycles, although both can affect microbial profiles.^23^, ^58^ Similarly, time between sample collection and processing, which introduces artifact to measures of relative abundance (eg, *Bacteroides* spp are selectively depleted at –80°C dependent on time in storage),^59^ was only explicitly mentioned by one study.^37^ Although microbiota characterisation through 16S rRNA gene amplicon sequencing allows bacterial determination robustly down to the genus level and is cost-effective, it is subject to bias associated with PCR primer-binding and bacterial taxonomic classification.^60^ Furthermore, the choice of reference database for bacterial taxonomic classification is crucial, and could lead to different results.^61^ In this systematic review, most studies used the Greengenes database; however, of note, this database was last updated in 2013.^62^ Use of an up-to-date reference database is important for accurate and high-resolution taxonomic assignment to enable comparisons of the sequencing output with a rapidly expanding and improving microbial genome taxonomy.^63^

A more informative genomic sequencing approach than the targeted amplicon sequencing method is shotgun metagenomic sequencing, which enables completely untargeted sequencing of all genetic material present in a stool sample; robust bacterial resolution down to a species or strain level; provides information about functionality; and detects viruses, archaea, fungi, and other microeukaryotes that do not possess the 16S rRNA gene.^19^, ^23^ Reporting microorganisms down to a strain or species taxa is crucial, as different species within the same genus can have different immunomodulatory effects.^64^

This systematic review has additional limitations. The search was restricted to studies published after 2010, and it is possible that some smaller studies were missed. One included study had important issues with reverse causality, and one study was a post-hoc analysis of a randomised controlled trial (RCT).^28^, ^34^ These studies did not meet the predetermined exclusion criteria but have important limitations and should be interpreted cautiously. Findings were summarised in two groups on the basis of outcome definitions, but alternative ways of grouping study findings could be used. As most studies did not report effect estimates, publication bias cannot be assessed with a funnel plot. However, the fact that some of the studies included in this systematic review reported null findings is somewhat reassuring.

Several frameworks have been developed to aid decisions in establishing causation in microbiome studies.^65^, ^66^ An important point in these frameworks is that inoculating a host and generating or preventing disease is a key step in providing evidence for causation.^20^, ^67^, ^68^ However, although some *Bifidobacterium* spp have been shown to influence respiratory disease pathogenesis in animal studies,^13^, ^53^ meta-analyses published between 2015 and 2020, including RCTs testing the efficacy of probiotics and prebiotics to prevent respiratory disease, have not shown a reduction in childhood risk of asthma, wheezing, or respiratory infections.^69^, ^70^, ^71^ Improving RCT study design and optimising observational studies to identify key bacterial species (most RCTs have focused on particular *Bifidobacterium* spp and *Lactobacillus* spp) for subsequently informing intervention studies,^70^ alongside optimal timing of intervention,^72^ is important.

Another important consideration is that the entire process of microbiota ecosystem development could be the cause of health or disease,^66^ rather than the absence or presence of specific microorganisms. The role of gut fungi, only explored in three studies,^29^, ^31^, ^37^ and gut viruses should be considered.^56^, ^72^ This concept has implications for future prevention strategies, potentially shifting the focus from introduction of single species towards designing probiotics with a rational mixture of species, or towards holistic interventions that might include changing perinatal clinical practice (eg, antibiotic use guidelines or diet).^18^, ^40^ However, such interventions might prove more complex to design, implement, and evaluate.

Another outstanding question when evaluating causality between gut microbiota and lung disease is the role of the respiratory microbiota.^57^ Given evidence of bidirectional influences between gut and respiratory microbiota,^73^ the potential effect of respiratory microbiota on the immune system, and the observed association between respiratory microbiota and childhood respiratory disease,^57^ respiratory microbiota might act as a confounder, mediator, or effect modifier in the association between gut microbiota and childhood respiratory disease.^57^ Longitudinal studies collecting both stool and respiratory samples might help to understand these complex interactions and elucidate the role of the gut–lung axis to identify targets for primary prevention interventions for childhood respiratory diseases.

Overall, there is observational evidence that low α-diversity and relative abundance of particular gut-commensal bacterial genera in the first year of life are associated with subsequent respiratory disease, especially asthma. There is less evidence for the association between low α-diversity and relative abundance of particular bacterial taxa in the first year of life and respiratory infections. However, the available evidence showed important limitations, and gut microbiota composition might not have a causal role in subsequent respiratory disease (despite the observed associations). Large longitudinal studies with stool and respiratory sampling during the neonatal period that use shotgun metagenomic sequencing to improve measurement resolution of the microbiome to a species or strain level are needed. Optimising statistical approaches for causal inference, standardising outcome definitions, and validating findings with experimental models will help move knowledge forward in the area of early-life microbiota and in the development of potential preventive and therapeutic interventions for childhood respiratory diseases.

## Declaration of interests

We declare no competing interests.
