# Supplementary material for: The association between early-life gut microbiota and childhood respiratory diseases: a systematic review
Source: Lancet Microbe. 2022 Nov;3(11):e867–80. doi: 10.1016/S2666-5247(22)00184-7 (PMC10499762; doi:10.1016/S2666-5247(22)00184-7)
Supplement: Supplementary appendix [file mmc1.pdf]

THE LANCET
Microbe

Supplementary appendix

This appendix formed part of the original submission and has been peer reviewed. We post it as supplied by the authors.

Supplement to: Alcazar C G-M, Paes VM, Shao Y, et al. The association between early-life gut microbiota and childhood respiratory diseases: a systematic review. *Lancet Microbe* 2022; published online Aug 18. https://doi.org/10.1016/S2666-5247(22)00184-7.

Supplementary material

Supplementary Table 1. Study selection criteria………………………………………………… 2

Supplementary Table 2. Search strategy…………………………………………………………. 3

Supplementary Table 3. Recruitment strategy, patient selection and study population…………….5

Supplementary Table 4. Other results related to respiratory disease outcome …………………...…8

Supplementary Table 5. Ottawa-Newcastle quality assessment scale for cohort studies…..……...11

Supplementary Table 6. Ottawa-Newcastle quality assessment scale for case-control studies………………………………………………………………………………………12

Supplementary Table 7. STROBE-metagenomics checklist……………………………………….14

References……………………………………………………………………………………….18

Supplementary Table 1. Study selection criteria

**Inclusion criteria**	○ Participants: children enrolled during the first year of life with follow-up to a maximum of 18 years of age<br>○ Exposure: gut microbiota composition measured at least once during the first year of life (0-12 months) using genomic sequencing methods<br>○ Outcome: any measure (questionnaire or clinical evaluation) during childhood (0-18 years) of acute respiratory infections, any form of wheezing, or asthma (hereon referred to as a combined outcome of 'respiratory disease')
**Exclusion criteria**	○ Reviews<br>○ Publications without original data<br>○ Book chapters<br>○ Conference abstracts<br>○ Clinical guidelines<br>○ Animal or in vitro studies<br>○ No access to full text in English<br>○ Cross-sectional study design<br>○ Fewer than 50 study participants<br>○ Study participants exclusively pre-term infants with <35 weeks gestational age<br>○ Gut microbiota composition measured only after one year of age

Supplementary Table 2. Search strategy

Embase			
Microbiome	Intestinal	Infancy	Respiratory disease
exp microbiome/ or exp bacterial microbiome/ or exp metagenome /or exp feces microflora/ or exp dysbiosis/ or exp intestine flora/ or exp biodiversity/ or actinobacteria/ or bacteroides/ or bifidobacterium/ or enterobacteriaceae/ or lactobacillus/ or proteobacteria/ or (microbiome or microbiota or dysbiosis or intestinal flora or biodiversity or actinobacteria or bacteroides or bifidobacterium or enterobacteriaceae or lactobacillus or proteobacteria).mp.	exp feces/ or exp intestine/ or meconium/ or (faeces or feces or meconium or intestin* or gut or stool).mp.	exp infant/ or exp baby/ or exp newborn/ or exp preschool child/ or (infant* or newborn* or baby or babies or neonate* or toddler* or preschool* or pre-school*).mp.	exp asthma/ or exp wheezing/ or exp bronchiolitis/ or exp viral bronchiolitis/ or exp Human respiratory syncytial virus/ or exp respiratory tract disease/ or exp respiratory tract infection/ (asthma* or wheezing or viral infection* or virus or infect* or bronchiolitis or bronchitis or respiratory or RSV or respiratory syncytial virus or pneumon* or bronchopneumon* or pleuropneumon).mp.
**Medline (via Ovid)**			
Microbiome	Intestinal	Infancy	Respiratory disease
exp Microbiota/ or exp Metagenome/ or exp Dysbiosis/ or exp Biodiversity/ or Actinobacteria/ or bacteroides/ or Bifidobacterium/ or Enterobacteriaceae/ or Lactobacillus/ or Proteobacteria/ or (microbiome or microbiota or dysbiosis or intestinal flora or biodiversity or actinobacteria or bacteroides or bifidobacterium or enterobacteriaceae or lactobacillus or proteobacteria).mp.	exp Feces/ or exp Meconium/ or exp Intestines/ or (faeces or feces or meconium or intestin* or gut or stool).mp.	exp Infant/ or exp Newborn/ or exp Child, Preeschool/ or (infant* or newborn* or baby or babies or neonate* or toddler* or preschool* or pre-school*).mp.	exp asthma/ or<br>exp Respiratory Sounds/ or<br>exp Bronchiolitis/ or exp Bronchiolitis, Viral/<br>exp Respiratory Tract Infections/ or exp Respiratory Syncytial Viruses/ or exp Respiratory Syncytial Virus Infections/ or exp pneumonia<br>(asthma* or wheezing or viral infection* or virus or infect* or bronchiolitis or bronchitis or respiratory or RSV or respiratory syncytial virus or pneumon* or bronchopneumon* or pleuropneumon).mp.

**Cochrane**			
Microbiome	Intestinal	Infancy	Respiratory disease
[mh Microbiota] or [mh Metagenome] or [mh Dysbiosis] or [mh Biodiversity] or [mh Actinobacteria] or [mh bacteroides] or [mh Bifidobacterium] or [mh Enterobacteriaceae] or [mh Lactobacillus] or [mh Proteobacteria] or (microbiome or	[mh Feces] or [mh Meconium] or [mh Intestines] or (faeces or feces or meconium or	[mh Infant] or [mh Newborn] or [mh Child, Preeschool] or (infant* or newborn* or baby or babies or neonate* or toddler* or	[mh asthma] or<br>[mh Respiratory Sounds] or<br>[mh Bronchiolitis] or [mh Bronchiolitis, Viral]<br>[mh Respiratory Tract Infections] or [mh Respiratory Syncytial Viruses]

microbiota or dysbiosis or intestinal flora or biodiversity or actinobacteria or bacteroides or bifidobacterium or enterobacteriaceae or lactobacillus or proteobacteria)	intestin* or gut or stool)	preschool* or pre-school*)	or [mh Respiratory Syncytial Virus Infections] or [mh Pneumonia] or (asthma or wheezing or viral infection* or virus or infect* or bronchiolitis or bronchitis or respiratory or RSV or respiratory syncytial virus or pneumon* or bronchopneumon* or pleuropneumon)
**Web of Science**			
Microbiome	Intestinal	Infancy	Respiratory disease
microbiome or microbiota or dysbiosis or intestinal flora or biodiversity or actinobacteria or bacteroides or bifidobacterium or enterobacteriaceae or lactobacillus or proteobacteria	faeces or feces or meconium or intestin* or gut or stool	infant* or newborn* or baby or babies or neonate* or toddler* or preschool* or pre-school*	asthma* or wheezing or viral infection* or virus or infect* or bronchiolitis or bronchitis or respiratory or RSV or respiratory syncytial virus or pneumon* or bronchopneumon* or pleuropneumon*
**Scopus**			
Microbiome	Intestinal	Infancy	Respiratory disease
microbiome or microbiota or dysbiosis or intestinal flora or biodiversity or actinobacteria or bacteroides or bifidobacterium or enterobacteriaceae or lactobacillus or proteobacteria	faeces or feces or meconium or intestin* or gut or stool	infant* or newborn* or baby or babies or neonate* or toddler* or preschool* or pre-school*	asthma* or wheezing or viral infection* or virus or infect* or bronchiolitis or bronchitis or respiratory or RSV or respiratory syncytial virus or pneumon* or bronchopneumon* or pleuropneumon*

Supplementary Table 3. Details of recruitment strategy, patient selection and study population

**ARRIETA** **et al. 2015**<br>NESTED CASE CONTROL ("CHILD" COHORT).<br>BIRTH-3 Y<br>**N= 319**<br>**(1)**	**Recruitment strategy:** CHILD birth cohort. Started enrolment in 2008 in 4 major Canadian cities. 3624 pregnant women enrolled and 3542 babies met eligibility criteria. Recruitment strategies ranged from having staff meet mothers in antenatal ultrasound clinics and physician offices to community "baby fairs" and included person-to-person referrals and social media advertising. Children were grouped in phenotypes (i) Atopic wheeze (AW) (ii) wheeze only (W)(iii) atopy only (A) (IV) controls (C). All children enrolled in CHILD with complete follow-up at 1 year (1427/3542; 40%) and available stool sample at both time points amongst the 4 phenotypes with adequate sequencing output were included in the study.<br>**Exclusion:** <35weeks, major congenital abnormalities or respiratory distress syndrome, expectation of moving away from a recruitment center within 1 year of recruitment, children of multiple births or resulting from in vitro fertilization, and children not spending over 80% of time in the index home.
**LAURSEN** **et al. 2015**<br>COHORT (SKOT 1)<br>9M-3Y<br>**N=114**<br>**(2)**	**Recruitment strategy:** Random selection of infants from the National Danish Civil Registry from 2007-2008.<br>**Inclusion:** Healthy, singleton term infants with an age of 9 months ±2 weeks.<br>**Exclusion:** Use of antibiotic at time of sampling or 7 days prior to sampling.
**FUJIMURA** **et al. 2016**<br>COHORT<br>BIRTH-4 Y<br>**N=298**<br>**(3)**	**Recruitment strategy:** From 2003-2007 the WHEALS cohort. Woman from 21-49 years recruited in 5 clinics of the Detroit area, in 2nd trimester of pregnancy. A total 1254 women recruited. Selection of infants: those with full follow-up at 24 months and dust samples collected at same time as the stool samples (n=308).<br>**Inclusion:** Mothers lived within an area of Detroit and spoke enough good English to sign the informed consent.
**STIEMSMA** **et al. 2016**<br>MATCHED. NESTED CASE-CONTROL. ("CHILD" COHORT)<br>BIRTH-4Y<br>**N=76**<br>**(4)**	**Recruitment strategy:** CHILD birth cohort. See Arrieta 2015 for enrolment. A total 3624 pregnant women enrolled, and 3542 babies met eligibility criteria. Only included children that had at least 3-year follow-up in the study (N=286/3542[8%]). Out of the 286 participants, cases and controls were selected.<br>**Exclusion:** <35weeks, major congenital abnormalities or respiratory distress syndrome (RDS), expectation of moving away from a recruitment center within 1 year of recruitment, children of multiple births or resulting from in vitro fertilization, and children not spending over 80% of time in the index home.
**ARRIETA** **et al. 2018**<br>NESTED CASE-CONTROL	**Recruitment strategy:** Between 2006 and 2009 in the public hospital serving the rural district of Quininde, Esmeraldas Province, a tropical region in Ecuador. Babies were recruited from the hospital maternal ward after delivery or vaccinations departments (research staff visited both). 2404 new-

("ECUAVIDA"<br>COHORT)<br>14D-5Y<br>**N=98**<br>**(5)**	borns recruited. Cases and controls were selected based on who had complete follow-up at 5 years (n=2090) and stool sample collected at 3 months (1066 stool samples).<br>**Inclusion:** Healthy baby less than 2weeks of age, at least one stool sample collected at time of delivery, the family lived in the district of Quininde for the last 2 years and did not plan to move out of the district over the following 3 years; the home was accessible; and the family had no ethical or religious principles that might interfere with their participation.
**STOCKHOLM et al. 2018**<br>COHORT<br>BIRTH-5Y<br>**N=690**<br>**(6)**	**Recruitment strategy:** Recruitment from 2008-1010 in Copenhagen, Denmark. Patients were contacted by mail and identified through GP pregnancy visits. Only those with a fecal sample (690/700).<br>**Inclusion:** Did not exclude preterm babies which represents 3% of the 690 cohort and does not mention comorbidities.<br>**Exclusion:** Gestational age above week 26 at time of recruitment or daily intake of more than 600 IU vitamin D during pregnancy.
**REYMAN et al. 2019**<br>COHORT<br>BIRTH-1Y<br>**N= 130**<br>(7)	**Recruitment strategy & patient selection**: Recruitment of babies born between 2012-2014 in Utrecht, Netherlands. Informed consent was obtained prenatally. Not specified how patients were enrolled. Patients selected if they had completed 1 year follow up and had at least five faecal samples available from ten possible timepoints.<br>**Inclusion:** Healthy, term babies (>37 weeks).<br>**Exclusion:** Major congenital anomalies, severe maternal or neonatal complications during birth, language barrier, intention to move outside the research area, or parents under 18 years of age.
**GALAZZO et al. 2020**<br>COHORT<br>(SECONDARY<br>ANALYSES RCT)<br>BIRTH-6-11Y<br>**N=440**<br>**(8)**	**Recruitment strategy & patient selection:** Recruitment from 2002-2007, in 32 hospitals in Berlin, Hamburg, Lower Saxony, and Brandenburg. Contacted parents from obstetrics department and new-borns enrolled within the 1st weeks of birth (n=606). From 5 weeks to 7 months babies were given either bacterial lysate containing heat-killed gram-negative *E. coli* Symbio and gram-positive *Enterococcus faecalis* Symbio or its placebo. Children with at least 3 stool samples collected during the first year and/or stool collected at school age (440/606).<br>**Inclusion:** Healthy new-borns at term, birth weight of 2500 g or greater, 1/2 parents with atopic disease (AD, allergic rhinitis, and/or asthma), and informed consent.<br>**Exclusion:** Treatment or other medication after birth, lymphocytopenia or thrombocytopenia, intensive care after birth, lack of German language knowledge or no informed consent.
**BOUTIN et al. 2020**<br>COHORT<br>("CHILD" COHORT)<br>BIRTH-5Y<br>**(9)**	**Recruitment strategy & patient selection:** CHILD birth cohort. See Arrieta 2015 for enrolment. A total 3624 pregnant women enrolled, and 3542 babies met eligibility criteria. A total 837 child participants had stool samples collected which were available for 16S. A total 659 had stool samples sequencing results & follow-up information at 1 year, 3 years and 5 years.<br>**Exclusion:** <35weeks, major congenital abnormalities or respiratory distress syndrome (RDS), expectation of moving away from a recruitment center within 1 year of recruitment, children of multiple births or resulting from in vitro fertilization, and children not spending over 80% of time in the index home.

**PATRICK** **et al. 2020**<br>("CHILD" COHORT)<br>BIRTH-6Y<br>N=917<br>**(10)**	**Recruitment strategy & patient selection:** CHILD birth cohort. See Arrieta 2015 for enrollment. A total 3624 pregnant women enrolled, and 3542 babies met eligibility criteria. A total 2644 clinically assessed for asthma at age 5years. Out of which 917 had sequencing data for stool samples collected at 3 months or/and 12 months.<br>**Exclusion:** <35weeks, major congenital abnormalities or respiratory distress syndrome, expectation of moving away from a recruitment center within 1 year of recruitment, children of multiple births or resulting from in vitro fertilization, and children not spending over 80% of time in the index home.
**DEPNER** **et al. 2020**<br>COHORT (PASTURE BIRTH COHORT)<br>N= 618<br>**(11)**	**Recruitment strategy:** Pregnant adult women were approached during 3rd trimester; 50% lived on family-run livestock farms. Children were recruited at birth. Recruitment happened from 2002-2005. A total 930 children recruited out of which 720 had sequencing data for stool samples collected at 2months and/or 12 months.<br>**Inclusion:** Families living in European rural areas from Austria, Finland, Germany and Switzerland.

n=number

Supplementary Table 4. Other results related to respiratory disease outcome (see Table 2)

AUTHOR/ YEAR	OTHER ANALYSES PERFORMED RELATED TO RESPIRATORY DISEASE	MAIN RESULTS
**ARRIETA et al. 2015**	(1) PICRUSt metagenomics for gut microbiota function (2) SCAFs 13 atopic wheeze (AW) samples and 13 controls; Urine metabolomics; LPS determination (3) Mice inoculation= Frozen feces (3m) from one AW subject used to orally inoculate GF mice. Then experimental murine allergic asthma was induced in all mice. Then OVA-specific IgE, IgG1, and IgG2a in serum were measured by enzyme-linked immunosorbent assay and lungs were looked at (pathology) and cytokines measured.	(1) Top 30 differential genes can discriminate between the AW group and controls at 3 months (not at 1 y). LPS biosynthesis was the pathway that differed most between both groups. (2) LPS concentration was lower in the faeces of AW children (Mann-Whitney, P = 0.09) and fecal acetate was significantly reduced in AW subjects at 3 months of age (p=0.03). (3) The microbiota from the AW sample induced a mixed T helper cell 1 (TH1) lung inflammatory response (measured by lung cytokines). Therapeutic colonization with FLVR significantly reduced the TH1/TH17 components of the immune response (p-value < 0.01 to 0.0001).
**LAURSEN et al. 2015**	n/a	n/a
**FUJIMURA et al. 2016**	(1) Mycobiome determination. Metabolomic profiling (2) Ex vivo dendritic cell challenge and T cell co-culture.	(1) Unsupervised clustering analyses (with DMM) of neonatal sample (NGM) showed three distinct groups (NGM 1, 2 and 3). NGM3, showed vs NGM1 and 2 depleted of multiple *Malassezia* taxa; and higher relative abundance of *Candida* and *Rhodotorula*, and a distinct fecal metabolome enriched for pro-inflammatory metabolites. (2) Ex vivo culture of human adult peripheral T cells with sterile fecal water from NGM3 subjects increased the proportion of CD4+ cells producing (IL)-4 and reduced the relative abundance of CD4+CD25+FOXP3+ cells. Conclusion: neonatal gut microbiome dysbiosis might promote CD4+ T cell dysfunction
**STIEMSMA et al. 2016**	*Lachnospira*/*C. neonatale* ratio to quantify dysbiosis and quartile analysis of the *Lachnospira/C. neonatale* ratio.	Quartile analysis of stool composition at 3 months showed a negative association between the ratio of these two bacteria (L/C) and asthma risk at 4y [quartile 1: odds ratio (OR) = 15, P = 0.02, CI (confidence interval) = 1.8–124.7; quartile 2: OR = 1.0, ns; quartile 3: OR = 0.37, ns]. Propose L/C ratio an early biomarker of asthma development.

**ARRIETA** et al. **2018**	(1) Mycobiome = diversity and relative abundance of species (adjusted for confounders); validation using qPCR (2) Differences in microbiome function: using PICRUSt (3) Differences in six most abundant SCFAs in faeces between atopic wheeze (AW) and controls.	(1) Fungal alterations were more marked than bacterial dysbiosis. In MaAslin: Greater relative abundance of fungi *Pichia Kudriavzevii* in AW vs controls. And validated increase in *Pichia* species using qPCR. (2) Taurine and hypotaurine metabolism, polyketide sugar unit biosynthesis, and carbohydrate digestion and absorption pathways were decreased in AWs (Welch t test) (3) significant decrease in acetate concentrations and an increase in caproate levels in the stool of babies in with atopic wheeze compared with controls.
**STOCKHOLM** et al. **2018**	(1) Cross-validated sparse PLS model to identify most important taxa for prediction of asthma. (2) PCA grouped population in 2 clusters mainly driven by age (PAM1 1w, 1m; PAM2 1y). Looked at association between transitioning from PAM1 to 2 or not, and development of asthma. (3) Maturity of the gut microbiota (MAZ) score (4) & (5) Analyses with asthma episodes (total number of asthma-like episodes in the first 3y). Potential problems with reverse causality.	(1) 60 different genera were identified with a prediction AUC 0.76: they conclude it's a global delayed microbial maturation at age 1 year that contribute to the subsequent asthma development. (2) Not transitioning from PAM1 to PAM2 was associated with later development of asthma, effect modification by maternal asthma (driven by those born to mothers with asthma). Adjusted for number of siblings. (3) MAZ at 1 year (low maturity, N = 257 vs. high maturity, N = 261) (HR 1.77 (1.02−3.07), P = 0.043). Low microbial maturity was only associated with later asthma in children born to asthmatic mothers (low maturity, N = 63 vs. high maturity, N = 57) (HR 6.53 (1.93−22.06), P=0.003), and not in children of non-asthmatic mothers (P = 0.81). Not clear if adjusted analyses. Not clear denominators. (4) In children born to asthmatic mothers AUC of 0.76 for predicting asthma at age 5 y from gut microbiota at 1y (5) Microbiota composition in PAM 1 at 1 y associated with > asthma episodes vs PAM 2 (PAM1 vs. PAM2: incidence risk ratio (IRR) 1.54 (1.12–2.12), P = 0.008). This was more pronounced in children born to asthmatic mothers. Lower maturity at 1 y (MAZ) associated with more episodes (IRR 1.13 (1.06–1.20), P < 0.001)
**REYMAN** et al. **2019**	(i)Random forest analysis was used to verify results (using respiratory infections (RI) events as outcome and the OTUs in the 1w samples as predictors, along with delivery mode and other clinical variables). These analyses were stratified	(i) *Enterococcus, Bifidobacterium,* and *Klebsiella* were verified as the most important taxa driving the prediction of the categorized RI events in 1y. (ii) The relative abundances of the top 12 OTUs and species of both sequencing methods show highly comparable profiles. qPCR

	for DM. (II)WGS (random sample of n=20) and qPCR to validate results and get information at a species level.	confirmed that colonization with Enterococcus spp. at 1w was positively associated with more RI events in the first year of life.
**GALAZZO** et al. **2020**	Maturity of the gut microbiota (MAZ) and its association with the development of asthma.	Higher microbial maturity at 5 weeks was associated with an increased risk for asthma (MAZ at 5 weeks OR adjusted 1.43; P= 7.78x10–3)
**PATRICK** et al. **2020**	Structural equational modelling	Microbiota composition (alpha and B-diversity measures) mediates the association between outpatient antibiotic use (1y) and increased asthma at 5y (adjusted estimate B=0·07, p=0·028)
**DEPNER** et al. **2020**	(1) Network analyses to determine specific protective of harmful taxa. Amplicon sequence variants (2) SCAF measurements were modeled using random forest (12m) in subsample of 209 children with a nested case-control study design. (3) Fungal age	(1) No specific taxa mas independently associated with asthma after adjustment for EMA except *Eggerthella* risk effect on asthma (OR:1.43 (1.07–1.92), P = 0.016) independently of EMA. Authors conclude that it may be the whole composition and adequate and timely maturity of the gut microbiota vs individual bacterial taxa that may protect against asthma. (2) Production of butyrate, propionate and acetate was most importantly predicted by *Roseburia, Bacteroides* and *Turicibacter*, respectively. Butyrate score was protective of asthma (OR: 0.38 (0.17–0.84), *P* = 0.017) (3) Fungal age was estimated similarly to EMA and was mainly determined by changes in: *Saccharomyces, Alternaria* and *Malassezia*, and was not associated with subsequent risk of asthma.

*Only reported if exposure was gut microbiota AW: atopic wheeze. GF: Germ free DM: delivery mode.AD: atopic dermatitis. DMM: Dirichlet multinomial mixtures; LPS: Lipopolysaccharide, FLVR: *Faecalibacterium, Lachnospira, Rothia and Veillonella,* SCAF: short chain fatty acids; PCA: Principal component analysis, PLS: Partial least squares, HR: Hazard ratio, AUC: Area under the curve, OTU: Operational taxonomic units, P=p=value, EMA: estimated microbiome age: random forest analysis (machine learning) were used to estimate the healthy age of gut microbiota sampled at 2m and 1y in n=133 'healthy' individuals (no diarrhea wheezing or asthma in the first 1y of life). WGS: whole genome sequencing.

Supplementary Table 5. Ottawa-Newcastle quality assessment scale for cohort studies

		Laursen et al. 2015	Fujimura et al. 2016	Stockholm et al. 2018	Reyman et al. 2019	Galazzo et al. 2020	Boutin et al. 2020	Patrick et al. 2020	Depner et al. 2020
**SELECTION**	1) Representativeness of the exposed cohort: a) truly representative of the average child in the community*; b) somewhat representative of the average child in the community*; c) selected group of users e.g. nurses, volunteers; d) no description of the derivation of the cohort	B	C	B	D	C	C	C	C
	2) Selection of the non-exposed cohort: a) drawn from the same community as the exposed cohort*; b) drawn from a different source; c) no description of the derivation of the non-exposed cohort	A	A	A	A	A	A	A	A
	3) Ascertainment of exposure (including confounding factors: a) secure record (e.g. clinical records)*; b) structured interview*; c) written self-report; d) no description	A/B	A/B	A/B	A/B	A/B	A/B	A/B	A/B
	4) Demonstration that outcome of interest was not present at start of study: a) yes*; b) no	B	A	A	A	A	A	A	A
**COMPARA BIITY**	1) Comparability of cohorts on the basis of the design or analysis:<br>a) study controls for BREASTFEEDING *<br>b) study controls for DELIVERY MODE *		X<br>X	X<br>X	 X	X<br>X		X<br>X	X<br>X
**OUTCOME**	1) Assessment of outcome: a) independent blind assessment*; b) record linkage*; c) self-report; d) no description	C	C	A	C	A	A	A	A
	2) Was follow-up long enough for outcomes to occur:<br>a) yes*; b) no	B	B	A	A	A	B	A	A
	3) Adequacy of follow up of cohorts: a) complete follow up - all subjects accounted for*; b) subjects lost to follow up unlikely to introduce bias - less than 20 % lost or description of those lost suggested no difference from those followed*; c) follow up rate < 80% and no description of those lost; d) no statement	B	C	B	B	C	C	C	B
	Total stars	4	5	9	6	7	4	7	8
	ONS scale converted to AHQR standards*	POOR	POOR	GOOD	GOOD	GOOD	POOR	GOOD	GOOD

Thresholds for converting the Newcastle-Ottawa scales to AHRQ standards (good, fair, and poor):
Good quality: 3 or 4 stars in selection domain AND 1 or 2 stars in comparability domain AND 2 or 3 stars in outcome domain
Fair quality: 2 stars in selection domain AND 1 or 2 stars in comparability domain AND 2 or 3 stars in outcome domain
Poor quality: 0 or 1 star in selection domain OR 0 stars in comparability domain OR 0 or 1 stars in outcome domain
Note: A study can be awarded a maximum of one star for each numbered item within the Selection and Outcome categories. A maximum of two stars can be given for Comparability

Supplementary Table 6. Ottawa-Newcastle quality assessment scale for case-control studies

		Arrieta et al. 2015	Stiemsma et al. 2016	Arrieta et al. 2018
**SELECTION**	1) Is the case definition adequate? a) yes, with independent validation * b) yes, e.g., record linkage or based on self-reports c) no description	A	A	B
	2) Representativeness of the cases a) consecutive or obviously representative series of cases * b) potential for selection biases or not stated	B	B	A
	3) Selection of Controls: a) community controls * b) hospital controls c) no description	A/B^	A/B^	B
	4) Definition of Controls: a) no history of disease (endpoint) * b) no description of source	A	A	A
**COMPARABIITY**	11) Comparability of cases and controls on the basis of the design or analysis			
	a) study controls for BREASTFEEDING *		X	X
	b) study controls DELIVERY MODE*		X	X
**EXPOSURE**	1) Ascertainment of exposure: a) secure record (eg surgical records) * b) structured interview where blind to case/control status * c) interview not blinded to case/control status d) written self-report or medical record only e) no description	A/B$	A/B$	A
	2) Same method of ascertainment for cases and controls a) yes * b) no	A	A	A
	3) Adequacy of follow up of cohorts (NESTED CASE-CONTROL): a) complete follow up - all subjects accounted for*; b) subjects lost to follow up unlikely to introduce bias - less than 20 % lost or description of those lost suggested no difference from those followed*; c) follow up rate < 80% and no description of those lost; d) no statement	C	C	B
	Total stars	5	7	7
	ONS scale converted to AHQR standards*	POOR	GOOD	FAIR

*Thresholds for converting the Newcastle-Ottawa scales to AHRQ standards (good, fair, and poor):
Good quality: 3 or 4 stars in selection domain AND 1 or 2 stars in comparability domain AND 2 or 3 stars in outcome domain
Fair quality: 2 stars in selection domain AND 1 or 2 stars in comparability domain AND 2 or 3 stars in outcome domain
Poor quality: 0 or 1 star in selection domain OR 0 stars in comparability domain OR 0 or 1 stars in outcome domain

Note: A study can be awarded a maximum of one star for each numbered item within the Selection and Outcome categories. A maximum of two stars can be given for Comparability

^ patients were recruited both from the community and hospital clinics $The two classifications aim to capture different methods of capturing exposure and clinical confounding variables.

Supplementary Table 7. STROBE-metagenomics checklist

		Arrieta et al 2015	Laursen et al. 2015	Fujimura et al. 2016	Stiemsma et al. 2016	Arrieta et al. 2018	Stockholm et al. 2018	Reyman et al. 2019	Galazzo et al. 2020	Boutin et al. 2020	Patrick et al. 2020	Depner et al. 2020
**Specimen collection, storage, and nucleic acid extraction methods**	Temperatures, and storage of samples.	Partial	Yes	Yes	Partial	Yes	Yes	Yes	Partial	Partial	Partial	Yes
	Detailed extraction methods	Yes	Yes	Yes	Yes	Yes	Yes	Yes	Referenced	Yes	Yes	Yes
	Filtration, centri-fugation, DNA digestion, rRNA depletion, separation in RNA or DNA, and random amplification.	Yes	Yes	yes	Yes	Yes	Yes	Yes	Yes	Yes	Yes	Yes
	Mentions use of standardized protocol	?	Yes	Yes	?	?	Yes	Yes	Yes	?	?	Yes
**Describe sequencing methods, including sequencing depth**	Mention sequencing platform	Yes	Yes	Yes	Yes	yes	Yes	Yes	Yes	Yes	Yes	Yes
	False positive and false negative errors mentioned as a limitation?	?	?	?	?	?	Yes	Yes	?	?	?	?
	Sequencing depth mentioned	?	Yes	Yes	?	Yes	Yes	Yes	Yes	Yes	Yes	Yes

	Reported: base calling, demultiplexing, trimming and removal of reads read Normalisation	Yes	Yes	Yes	Yes	Yes	Yes	Yes	Yes	Yes	Yes	Yes
	Software name, version, main commands	Yes	Yes	Yes	Yes	Yes	Yes	Yes	Yes	Yes	Yes	Yes
	Data and programming code open access?	?	Yes	?	?	?	Yes	Yes	?	?	?	Yes
**Quality assurance methods**	Internal controls reported as part of standard operating procedures	Yes	?	?	Yes	Yes	Yes	Yes	?	Yes	Yes	?
**Orthogonal methods to confirm pathogen identity, function and viability**	Confirmatory assays appropriate to the study setting,	Yes (qPCR)	?	?	Yes (qPCR)	?	?	Yes (qPCR)	?	?	?	? (measures SCAFs)
**Describe the criteria used to assess the role of pathogens in disease aetiology.**	Temporality	Problem with 1 year sample.	Problem (reverse causality)	Ok	Ok	Ok	Ok for main analyses.	OK	OK	OK	OK	OK
	Experimental (if they inoculate pathogens)	Yes (mouse model)	?	Yes (faecal samples)	?	?	?	?	?	?	?	?
**State the time from collection to results and**	Time from sample collection to processing reported	?	Yes (partial)	?	?	?	?	?	?	?	?	Yes

**cost consideration**												
	Sequencing run time and total computational analysis time reported	?	?	?	?	?	?	?	?	?	?	?
**Setting and patient recruitment**	State whether sample collection was retrospective or prospective	Yes	Yes	Yes	Yes	Yes	Yes	Yes	Yes	Yes	Yes	Yes
	Analyses adjusted/matched for confounding factors	?	?	Yes	Yes	Yes	Yes	Yes	Yes	?	Yes	Yes
**Addressed potential bias introduced by bioinformatics analysis**	Rarefaction step performed to deal with variation in read counts (Sophisticated statistical modelling approaches to deal with variation in read numbers between samples without loss of data)	Yes (Mothur)	N/A	Yes (QIIME)	Yes (Mothur)	Yes (Mothur or QIIME)	Yes (Mothur)	Yes (QIIME)	?	Yes (QIIME2)	Yes (QIIME2)	Yes (QIIME2)
**Describe or address limitations of reference databases**	Reference database stated	Yes	Yes	Yes	Yes	Yes	Yes	Yes	Yes	Yes	Yes	Yes
**Study size and Statistical methods**	Effect size reported	?	?	Yes (by DMM group)	Yes (by quartiles, not in main analyses for review)	?	Yes	Yes (not for main analysis)	Yes (not for review question analysis)	Yes (only for diversity)	Yes	Yes

	Number of comparisons/methods used to correct for multiple comparisons	Yes	Yes	Yes	Yes	Yes	Yes	Yes	Yes	?	Yes	?
	Details of the statistical methods used for power calculations reported or mentioned	Mentioned but not calculated	? (mentioned power but not calculated)	? (not mentioned for review question)	Yes (post/hoc).	?	?	Yes	?	?	?	?
	Limit of detection mentioned	Yes	Yes	Yes	Yes	Yes	Yes	Yes	Yes	Yes	Yes	Yes
	STROME-ID (12) state how the study dealt with missing data	?	?	?	Yes	?	?	?	Yes	?	Yes	Yes (reported missing data)

DMM: Dirichlet multinomial mixtures, SCAFs: short chain fatty acids

REFERENCES

1. Arrieta MC, Stiemsma LT, Dimitriu PA, Thorson L, Russell S, Yurist-Doutsch S, et al. Early infancy microbial and metabolic alterations affect risk of childhood asthma. Science Translational Medicine. 2015;7(307):307ra152.
2. Laursen MFZ, G.: Bahl, M. I.: Bergstrom, A.: Host, A.: Michaelsen, K. F.: Licht, T. R. Having older siblings is associated with gut microbiota development during early childhood. BMC Microbiology. 2015;15:154.
3. Fujimura KE, Sitarik AR, Havstad S, Lin DL, Levan S, Fadrosh D, et al. Neonatal gut microbiota associates with childhood multisensitized atopy and T cell differentiation. Nature Medicine. 2016;22(10):1187-91.
4. Stiemsma LT, Arrieta MC, Dimitriu PA, Cheng J, Thorson L, Lefebvre DL, et al. Shifts in Lachnospira and Clostridium sp. in the 3-month stool microbiome are associated with preschool age asthma. Clinical Science. 2016;130(23):2199-207.
5. Arrieta MC, Arevalo A, Stiemsma L, Dimitriu P, Chico ME, Loor S, et al. Associations between infant fungal and bacterial dysbiosis and childhood atopic wheeze in a nonindustrialized setting. Journal of Allergy & Clinical Immunology. 2018;142(2):424-34.e10.
6. Stokholm J, Blaser MJ, Thorsen J, Rasmussen MA, Waage J, Vinding RK, et al. Maturation of the gut microbiome and risk of asthma in childhood. Nature communications. 2018;9(1):141.
7. Reyman MvH, M. A.: van Baarle, D.: Bosch, Aatm: Man, W. H.: Chu, Mljn: Arp, K.: Watson, R. L.: Sanders, E. A. M.: Fuentes, S.: Bogaert, D. Impact of delivery mode-associated gut microbiota dynamics on health in the first year of life. Nature communications. 2019;10(1):4997.
8. Galazzo GvB, N.: Bervoets, L.: Dapaah, I. O.: Savelkoul, P. H.: Hornef, M. W.: Gi-Mdh consortium: Lau, S.: Hamelmann, E.: Penders, J.: Gi-Mdh consortium: Hutton, E. K.: Morrison, K.: Holloway, A. C.: McDonald, H.: Ratcliffe, E. M.: Stearns, J. C.: Schertzer, J. D.: Surette, M. G.: Thabane, L.: Mommers, M. Development of the Microbiota and Associations With Birth Mode, Diet, and Atopic Disorders in a Longitudinal Analysis of Stool Samples, Collected From Infancy Through Early Childhood. Gastroenterology. 2020;17:17.
9. Boutin RCT, Sbihi H, Dsouza M, Malhotra R, Petersen C, Dai D, et al. Mining the infant gut microbiota for therapeutic targets against atopic disease. Allergy: European Journal of Allergy and Clinical Immunology. 2020;75(8).
10. Patrick DM, Sbihi H, Dai DLY, Al Mamun A, Rasali D, Rose C, et al. Decreasing antibiotic use, the gut microbiota, and asthma incidence in children: evidence from population-based and prospective cohort studies. The Lancet Respiratory Medicine. 2020;8(11):1094-105.
11. Depner M, Taft DH, Kirjavainen PV, Kalanetra KM, Karvonen AM, Peschel S, et al. Maturation of the gut microbiome during the first year of life contributes to the protective farm effect on childhood asthma. Nature Medicine. 2020;26(11):1766-75.
12. Bharucha T, Oeser C, Balloux F, Brown JR, Carbo EC, Charlett A, et al. STROBE-metagenomics: a STROBE extension statement to guide the reporting of metagenomics studies. Lancet Infect Dis. 2020.
